# The effects of a low international normalized ratio on thromboembolic and bleeding complications in patients with mechanical mitral valve replacement

**DOI:** 10.1186/1749-8090-9-79

**Published:** 2014-05-07

**Authors:** Ugur Bal, Alp Aydinalp, Kerem Yilmaz, Emre Ozcalik, Senem Hasirci, Ilyas Atar, Bahadir Gultekin, Atilla Sezgin, Haldun Muderrisoglu

**Affiliations:** 1 Department of Cardiology, Baskent University School of Medicine, Fevzi Cakmak Caddesi 10. Sokak No: 45, Bahcelievler 06490, Ankara, Turkey; 2 Department of Cardiothoracic Surgery, Baskent University School of Medicine, Ankara, Turkey

**Keywords:** Mechanical heart valve, Anticoagulation, INR, Mitral valve replacement

## Abstract

**Background:**

Mechanical heart valve replacement has an inherent risk of thromboembolic events (TEs). Current guidelines recommend an international normalized ratio (INR) of at least 2.5 after mechanical mitral valve replacement (MVR). This study aimed to evaluate the effects of a low INR (2.0–2.5) on thromboembolic and bleeding complications in patients with mechanical MVR on warfarin therapy.

**Methods:**

One hundred and thirty-five patients who underwent mechanical MVR were enrolled in this study. The end points of this study were defined as TEs (valve thrombosis, transient ischemic attack, stroke) and bleeding (all minor and major bleeding) complications. Patients were followed up for a mean of 39.6 months and the mean INR of the patients was calculated. After data collection, patients were divided into 3 groups according to their mean INR, as follows: group 1 (n = 34), INR <2.0; group 2 (n = 49), INR 2.0–2.5; and group 3 (n = 52), INR >2.5.

**Results:**

A total of 22 events (10 [7.4%] thromboembolic and 12 [8.8%] bleeding events) occurred in the follow-up period. The mean INR was an independent risk factor for the development of TEs. Mean INR and neurological dysfunction were independent risk factors for the development of bleeding events. A statistically significant positive correlation was found between the log mean INR and all bleeding events, and a negative correlation was found between the log mean INR and all TEs. The total number of events was significantly lower in group 2 than in groups 1 and 3 (*P* = 0.036).

**Conclusions:**

This study showed that a target INRs of 2.0–2.5 are acceptable for preventing TEs and safe in terms of bleeding complications in patients with mechanical MVR.

## Background

Lifelong oral anticoagulation is recommended for all patients with mechanical heart valves irrespective of valve type or date of introduction and is essential for the prevention of thromboembolic events (TEs) [1,2]. However, anticoagulation therapy is also associated with an increased risk of bleeding complications [3]. It is important to achieve optimum anticoagulation, which prevents both adverse thromboembolic and bleeding complications. Although current clinical guidelines—European and American—provide evidence-based recommendations on optimum anticoagulation, there is disagreement about the thrombogenicity of mechanical heart valves and tailoring of anticoagulation goals [4,5]. These guidelines recommend higher international normalized ratios (INRs) due to the higher rates of thromboembolic complications of the prosthesis in the mitral position than in the aortic position. A target INR range of 2.5–4.0 is the current recommendation for patients with mechanical mitral valve replacement (MVR) [4,5].

Many factors may contribute to poor anticoagulant control, including patient compliance, lifestyle, diet, drug interactions and irregular access to controls. Therefore, it may be difficult to achieve the target INR with vitamin-K antagonists such as warfarin, and most patients do not achieve the target INR. Although previous studies suggest that a low INR is preferable and safe for mechanical heart valves, the effect of a low INR in patients with mechanical MVR alone has not been studied [6-12]. This study aimed to evaluate the effect of a low INR (2.0–2.5) on thromboembolic and bleeding complications in patients with mechanical MVR on warfarin therapy.

## Materials and methods

All patients who underwent solely mechanical MVR between 2004 and 2012 at the Baskent University Hospital were considered for inclusion in the study. One hundred and fifty-nine patients who underwent mechanical MVR were enrolled in the study. All patients were required to be present for outpatient assessments, twice in the first 3 months, and monthly in the remaining time after hospital discharge. Patients (n = 24) who were not regular outpatients with measured IRNs after valve surgery were excluded. All patients were informed about the effects of dietary habits on the coagulation parameters and used the same source of warfarin sodium.

The same warfarin therapy was administered to all patients for anticoagulation. To evaluate anticoagulant activity, the INRs were measured every month and a mean INR was calculated for each patient. All patients were informed of the effects of dietary habits on the coagulation parameters. The presence of bleeding and thromboembolic complications was recorded. Bleeding episodes were classified as major, minor, and minimal as described in the Thrombolysis in Myocardial Infarction (TIMI) trial [13]. Minimal bleeding (gingival bleeding, ecchymosis or other episodes of simple bleeding) was not included in the statistical analysis. Thromboembolism was defined as a transient or permanent neurological, limb or visceral deficit.

After data collection, patients were divided into 3 groups, as follows: group 1, the patients with mean INR <2.0 (n = 34) that we were unable to achieve a target INR level attributed to numerous factors including change in diet, poor compliance with medication, and low socio-cultural level of the patient, alcohol consumption, seasonal variation, and drug to drug interactions; group 2, mean INR 2.0–2.5 (n = 49); and group 3, mean INR >2.5 (n = 52). The main end points of the study were defined as thromboembolic (valve thrombosis, transient ischemic attack and stroke) and bleeding (all minor and major bleeding) events.

This study has been approved by the Baskent University Institutional Review Board and Ethics Committee, and supported by the Baskent University Research Fund.

### Statistical analysis

The statistical package SPSS (Statistical Package for the Social Sciences, version 17.0, SSPS Inc, Chicago, Ill, USA) was used for statistical analysis. Continuous variables were expressed as means ± standard deviation. Categorical variables were expressed as the total number (percentage). All continuous variables were evaluated with the Kolmogorov–Smirnov normality test to show their distributions. Continuous variables with normal distributions were compared using the unpaired Student *t*-test and ANOVA with the Tukey’s post-hoc test. Continuous variables with abnormal distributions were compared using the Mann–Whitney *U* test and ANOVA with the Tukey’s post-hoc test. For categorical variables, the chi-square test was used. Values of *P* < 0.05 were considered statistically significant for all tests.

The relationship between the mean INR and thromboembolic and bleeding events were examined by Pearson’s correlation analysis.

A multiple regression analysis was performed to examine the independent predictors of bleeding and TEs. The alternative test hypothesis was built as two-sided for each statistical analysis. The tests were independent and so was the experiment-wise. Type I error did not exceed 0.05 alpha levels. Univariate variables with *P* < 0.05 were included in the multiple regression analysis for odds ratios and 95% confidence intervals.

## Results

### Patients’ characteristics

Fifty (37%) of the patients were men and 85 (63%) were women with a mean age of 51.7 ± 13.7 years. The duration of the follow-up period was 12–72 months (mean 39.6 ± 21.4 months), with a cumulative follow-up period of 446.2 patient-years. Initial mitral valve dysfunction was attributed to rheumatic mitral stenosis in 66.7% (n = 90) of cases and to other causes such as functional mitral regurgitation or chordae rupture in the remaining 33.3% (n = 45) of the cases. The reason for undergoing surgery was similar in all groups. The baseline clinical characteristics and laboratory parameters of the patients are shown in Table 1.

**Table 1:** The baseline clinical characteristics and laboratory parameters of the patients

**Variables**	**Group 1 n = 34**	**Group 2 n = 49**	**Group 3 n = 52**	*F	*p
Age, years	55.9 ± 12.9	49.9 ± 13.2	50.1 ± 14.2	2.428	0.092
Gender, male (%)	23 (67.6)	33 (67.3)	30 (57.7)	-	0.516
Atrial fibrillation, n (%)	17 (50)	28 (57.1)	26 (50)	-	0.727
Hypertension, n (%)	16 (47.1)	14 (28.6)	19 (36.5)	-	0.227
Diabetes mellitus, n (%)	8 (23.5)	5 (10.2)	4 (7.7)	-	0.079
Smoking, n (%)	5 (20)	15 (32.6)	11 (21.6)	-	0.360
Neurological dysfunction, n (%)	3 (8.8)	3 (6.1)	2 (3.8)	-	0.632
Follow-up time, months	33.7 ± 29.0	40.4 ± 32.9	42.8 ± 31.5	0.884	0.416
Mean LVEF, %	53.3 ± 9.8	53.2 ± 8.8	54.0 ± 9.2	0.778	0.461
Left atrium, cm	5.2 ± 0.8	5.8 ± 1.0	5.5 ± 1.0	3.727	**0.027**
sPAP, mmHg	40.8 ± 12.7	43.8 ± 14.7	42.2 ± 12.5	0.506	0.604
Hemoglobin, g/dL	13.6 ± 1.5	12.7 ± 1.6	12.3 ± 1.3	7.899	**0.001**
WBC, ×10^3^/μL	7.8 ± 2.7	8.5 ± 2.2	7.7 ± 1.9	2.098	0.127
Platelet, ×10^3^/μL	196.91 ± 68.1	218.98 ± 77.7	231.77 ± 78.4	2.181	0.117
Creatinine, mg/dL	1.24 ± 1.3	1.39 ± 1.5	0.9 ± 0.2	2.201	0.115
Mean INR level	1.8 ± 0.1	2.2 ± 0.1	2.8 ± 0.3	232.346	**<0.001**

INR: International Normalized Ratio, LVEF: Left Ventricle Ejection Fraction, sPAP: Systolic Pulmonary Arterial Pressure, WBC: White Blood Cell.

*Chi-square and ANOVA with posthoc Tukey test.

A total of 22 thromboembolic and bleeding events were documented in the follow-up period (Table 2).

**Table 2:** Main outcomes of the patients

**Variable**	**Total n = 135**	**Group 1 n = 34**	**Group 2 n = 49**	**Group 3 n = 52**	**P value**
**Thromboembolic Events, n (%)**	10 (7.4)	7 (20.5)	1 (2.0)	2 (3.9)	**0.003**
Valve Thrombosis, n (%)	2 (1.4)	2 (5.8)	0	0	**0.049**
Stroke, n (%)	4 (2.9)	4 (11.7)	0	0	**0.002**
TIA, n (%)	4 (2.9)	1 (2.9)	1 (2.0)	2 (3.8)	0.867
**Bleeding Events, n (%)**	12 (8.8)	2 (5.8)	2 (4.0)	8 (15.3)	0.106
Major, n (%)	4 (2.9)	1 (2.9)	0	3 (5.8)	0.232
Minor, n (%)	8 (5.9)	1 (2.9)	2 (4.0)	5 (9.6)	0.348
**Total Events, n (%)**	22 (16.2)	9 (26.4)	3 (6.1)	10 (19.2)	**0.036**

TIA: Transient Ischemic Attacks.

Chi-square test.

### Predictors of thromboembolic events

The incidence of TEs (10 events were documented [7.4%]: group 1, n = 7 [20.5%]; group 2, n = 1 [2.0%]; group 3, n = 2 [3.8%]) was significantly higher in group 1 (*P* = 0.003) than in the other 2 groups. Four (2.9%) of the TEs were transient ischemic attacks (TİA), (group 1, n = 1 [2.9%]; group 2, n = 1 [2.0%]; group 3, n = 2 [3.8%]) and there was no significant difference among the 3 groups regarding TİAs (p = NS). All cases of ischemic stroke (n = 4, 11.7%) and stuck valve (n = 2, 5.8%) were reported in group 1 with significantly higher incidence than that in the other 2 groups ((P = 0.002 and P = 0.049, respectively; Table 2).

In the univariate analysis, we found that the mean INR was significantly lower in patients with TEs than in patients without TEs (*P* = 0.022) and the other parameters were similar among the groups (Table 3). Multiple stepwise logistic regression analysis showed that the mean INR (OR, 9.77; 95% CI, 1.26–75.52; *P* = 0.029) was an independent risk factor for the development of TEs in this patient group.

**Table 3:** Univariate analysis for presence of any thromboembolic or bleeding events

	**Patients with TEs N: 10**	**Patients without TEs N: 125**	**P value**	**Patients with bleeding events N: 12**	**Patients without bleeding events N: 123**	**P value**
Age, years	49.3 ± 13.8	51.7 ± 13.7	0.600	48.0 ± 9.1	51.8 ± 14.1	0.356
Gender, male (%)	4 (40.0)	45 (36.0)	1.0	4 (33.3)	45 (36.6)	1.0
Atrial fibrillation, n (%)	8 (80)	63 (50.4)	0.101	7 (58.3)	64 (52)	0,768
Hypertension, n (%)	5 (50)	44 (35.2)	0.496	4 (33.3)	45 (36.6)	1.0
Diabetes mellitus, n (%)	0	17 (13.6)	0.361	1 (8.3)	16 (13)	1.0
Smoking, n (%)	3 (37.5)	28 (24.6)	0.418	4 (33.3)	27 (24.5)	0.498
Neurological dysfunction, n (%)	2 (20)	6 (4.8)	0.109	3 (25)	5 (4.1)	**0.024**
Follow-up Time, months	47.1 ± 30.7	39.0 ± 30.5	0.439	53.2 ± 39.4	38.3 ± 30.4	0.117
Mean LVEF, %	55.9 ± 10.2	53.9 ± 9.1	0.514	54.0 ± 12.9	54.0 ± 8.9	0.984
Left Atrium, cm	5.6 ± 1.1	5.5 ± 1.0	0.799	5.7 ± 0.9	5.5 ± 1.0	0.642
sPAP, mmHg	44.1 ± 12.9	42.3 ± 13.5	0.692	41.9 ± 12.5	42.5 ± 13.5	0.881
Hemoglobin, g/dL	12.7 ± 1.1	13.0 ± 1.6	0.512	13.2 ± 1.4	13.0 ± 1.7	0.587
Platelet, ×10^3^/μL	211 ± 63	218 ± 77	0.769	259 ± 57	214 ± 76	0.051
Creatinine, mg/dL	0.8 ± 0.2	1.2 ± 1.2	0.283	0.9 ± 0.2	1.2 ± 1.2	0.457
Mean INR level	2.1 ± 0.4	2.4 ± 0.5	**0.022**	2.4 ± 0.4	2.7 ± 0.5	**0.016**

INR: International Normalized Ratio, LVEF: Left Ventricle Ejection Fraction, sPAP: Systolic Pulmonary Arterial Pressure, TEs: Thromboembolic events.

Data are presented as number (percentage) and mean ± SD values.

Chi-square, unpaired Student-t test and the Mann–Whitney U test.

### Predictors of bleeding events

A total of 12 bleeding episodes were documented in the follow-up period (8.8%; group 1, n = 2 [5.8%]; group 2, n = 2 [4.0%]; group 3, n = 8 [15.3%]). The bleeding events were not significantly different between the groups (*P* = 0.106). There were 4 major (2.9%; group 1, n = 1 [2.9%]; group 2, n = 0 [0%]; group 3, n = 3 [5.8%]) and 8 minor (5.9%; group 1, n = 1 [2.9%]; group 2, n = 2 [4.0%], group 3, n = 5 [9.6%]) bleeding events. Three of the major bleeding events were documented in group 3 (5.7%; one intracranial hemorrhage and two gastrointestinal system bleeding events) and the other major bleeding event (intracranial hemorrhage) was documented in group 1. The patient with intracranial hemorrhage in group 3 died. There were 5 minor bleeding episodes in group 3.

In the univariate analysis, we found that the mean INR and neurological dysfunction (which was already being due to a TE and present prior to undergoing valve surgery and initiating anticoagulation) were significantly higher in patients with bleeding events than in patients without bleeding events (*P* = 0.016 and *P* = 0.024, respectively), and the other parameters were similar in the 3 groups (Table 3). Multiple stepwise logistic regression analysis showed that the mean INR (OR, 5.26; 95% CI, 1.41–19.6; *P* = 0.013) and neurological dysfunction (OR, 11.7; 95% CI, 1.99–68.5; *P* = 0.006) were independent risk factors for the development of bleeding events in this patient group.

### Correlation between mean INR and thromboembolic or bleeding events

Significant positive correlations were found between the log mean INR and all bleeding events (*r* = 0.206, *P* = 0.016). Negative correlations were found between the log mean INR and all TEs (*r* = -0.197, *P* = 0.022; Table 4).

**Table 4:** Correlation between of mean INR levels and thromboembolic or bleeding events

	**All patients**	
	***r***	***p***
All Thromboembolic Events	-0.197	**0.022**
Valve Thrombosis	-0.147	0.089
Stroke	-0.162	0.060
TIA	-0.037	0.673
All Bleeding Events	0.206	**0.016**
Major Bleeding	0.144	0.096
Minor Bleeding	0.146	0.092
Total Events	-0.020	0.822

TIA: Transient Ischemic Attacks.

Pearson correlation analysis.

## Discussion

We studied the INRs in patients undergoing mechanical MVR, without interference with the surgeries conducted by the treating physicians. Relatively stable drug doses were administered. A total of 135 patients with mechanical MVR were followed up, and we did not observe any significant differences in the incidence of TEs between patients with INRs of 2.0–2.5 and those with INRs >2.5. Moreover, the number of events was significantly lower in patients with INRs of 2.0–2.5 and this patient group was the safest group in our study.

Lifelong anticoagulation is essential for all patients with mechanical heart valve prostheses. However, it is difficult to achieve optimal anticoagulation with warfarin, which requires a careful balance. Low INR may result in thromboembolic complications. In contrast, high INR may result in bleeding complications such as intracranial hemorrhages or gastrointestinal bleeding [2]. Stein at al. [14] found that the rate of morbidity from bleeding (7% per patient year) was higher than that from thromboembolism (1–2% per patient year). They concluded that anticoagulation should be controlled within an ideal range to minimize bleeding complications.

Our results are consistent with those of other studies from Asia. Mori et al. [6] studied 102 Japanese patients with mechanical heart valve prosthesis and found a significant increase in morbidity from bleeding with an INR >2.5. Uetsuka et al. [7] studied 1,157 Japanese patients receiving warfarin therapy after prosthetic valve replacement during a mean follow-up period of 2.44 years and found that the incidence of TEs did not increase with a lower INR of 1.5. Therefore, they suggested that the incidence of TEs is lower in Japan, despite a less intensive regimen.

From China, Sun et al. [8] studied 805 patients with St. Jude Medical valves and concluded that a lower target INR range of 2.0–2.5 is preferable for Chinese patients to reduce severe bleeding complications in those with conventionally higher INRs. Zhang et al. [9] studied 1,658 patients who underwent mechanical valve replacement and reported that the incidence of total complications in the group with INRs of 1.3–2.3 (aortic valve replacement: 1.3–1.8; mitral valve replacement and double valve replacement: 1.8–2.3) was the lowest among all anticoagulant therapy regimens followed.

In a prospective study from Pakistan, Raja et al. [10] reported that anticoagulation for mechanical heart valve replacement can be managed with a target INR range of 2.0–2.5, resulting in acceptable hemorrhagic and thromboembolic events.

In western countries, Koertke et al. [11] followed 1,137 German patients in a prospective, randomized, multicenter trial and demonstrated the efficacy and safety of very low, self-managed INR doses (INR target value, 2.0; range, 1.6–2.1) in patients with aortic valve replacement and 2.3 (range, 2.0–2.5) in patients with mitral valve or double valve replacement. In France, the AREVA study reported that the incidence of thromboembolic complications was similar in patients with a target INR range of 2.0–3.0 and those with a target INR range of 3.0–4.5; however, there were fewer bleeding complications in the low dose group [12].

We also found that neurological dysfunction was an independent risk factor for bleeding events. We have attributed this finding to the decreased cognitive function and physical disability of patients with neurological dysfunction. Moreover, the inability to reach an optimal medical control was another factor for bleeding complications in this patient group.

Socio-economic circumstances have a great role in the management and compliance of patients with mechanical MVR. Thus, the small number of patients with optimal INR levels can be explained by the higher rates of patients with a low socio-economic status in this study.

## Study limitations

The small number of the subjects is the major limitation of this study.

## Conclusion

This study showed that a target INR range of 2.0–2.5 is acceptable for preventing TEs and safe in terms of bleeding complications in patients with mechanical MVR. In the future, population-based INR adjustments can be preferred to continental guidelines for valve replacement patients; but however, large local studies are needed to allow changes in current guidelines.

## Abbreviations

TEs: Thromboembolic events; MVR: Mitral valve replacement; INR: International normalized ratio; TİA: Transient ischemic attacks.

## Competing interests

The authors declare that they have no competing interests.

## Authors’ contributions

UB participated in the design of the study and draft the manuscript. AA conceived of the study and participated in its design and coordination. KCY participated in acquisition of data and coordination. EO and SHH participated in acquisition of data. IA participated in the design of the study and performed the statistical analysis. BG participated in coordination and helped to draft the manuscript. AS participated in study design and coordination and helped to draft the manuscript. HM participated in revising the study critically for important intellectual content. All authors read and approved the final manuscript.

## References

[B1] Salem DN Stein PD Al-Ahmad A Bussey HI Horstkotte D Miller N Pauker SG Antithrombotic therapy in valvular heart disease–native and prosthetic: the Seventh ACCP Conference on Antithrombotic and Thrombolytic Therapy Chest 2004 9 457 82 10.1378/chest.126.2.457 15383481

[B2] Cannegieter SC Rosendaal FR Briët E Thromboembolic and bleeding complications in patients with mechanical heart valve prostheses Circulation 1994 9 635 41 10.1161/01.CIR.89.2.635 8313552

[B3] Levine MN Raskob G Beyth RJ Kearon C Schulman S Hemorrhagic complications of anticoagulant treatment: the Seventh ACCP Conference on Antithrombotic and Thrombolytic Therapy Chest 2004 9 287S 310S 10.1378/chest.126.3_suppl.287S 15383476

[B4] Guidelines on the management of valvular heart disease (version 2012) The Joint Task Force on the Management of Valvular Heart Disease of the European Society of Cardiology (ESC) and the European Association for Cardio-Thoracic Surgery (EACTS) Eur Heart J 2012 9 2451 96 22922698 10.1093/ejcts/ezs455

[B5] Bonow RO Carabello BA Chatterjee K de Leon AC Jr Faxon DP Freed MD Gaasch WH Lytle BW Nishimura RA O'Gara PT O'Rourke RA Otto CM Shah PM Shanewise JS 2008 focused update incorporated into the ACC/AHA 2006 guidelines for the management of patients with valvular heart disease: a report of the American College of Cardiology/American Heart Association Task Force on Practice Guidelines (Writing Committee to revise the 1998 guidelines for the management of patients with valvular heart disease). Endorsed by the Society of Cardiovascular Anesthesiologists, Society for Cardiovascular Angiography and Interventions, and Society of Thoracic Surgeons J Am Coll Cardiol 2008 9 13 e1 142 10.1016/j.jacc.2008.05.007 18848134

[B6] Mori T Asano M Ohtake H Bitoh A Sekiguchi S Matsuo Y Aiba M Yamada M Kawada T Takaba T Anticoagulant therapy after prosthetic valve replacement-optimal PT-INR in Japanese patients Ann Thorac Cardiovasc Surg 2002 9 83 7 12027793

[B7] Uetsuka Y Hosoda S Kasanuki H Aosaki M Murasaki K Ooki K Inoue M Akiyama E Kitada M Optimal therapeutic range for oral anticoagulants in Japanese patients with prosthetic heart valves: a preliminary report from a single institution using conversion from thrombotest to PT-INR Heart Vessels 2000 9 124 8 10.1007/PL00007265 11289500

[B8] Sun X Hu S Qi G Zhou Y Low standard oral anticoagulation therapy for Chinese patients with St. Jude mechanical heart valves Chin Med J 2003 9 1175 8 12935405

[B9] Haibo Z Jinzhong L Yan L Xu M Low-intensity international normalized ratio (INR) oral anticoagulant therapy in Chinese patients with mechanical heart valve prostheses Cell Biochem Biophys 2012 9 147 51 10.1007/s12013-011-9275-4 21892782

[B10] Akhtar RP Abid AR Zafar H Khan JS Anticoagulation in patients following prosthetic heart valve replacement Ann Thorac Cardiovasc Surg 2009 9 10 7 19262444

[B11] Koertke H Zittermann A Wagner O Ennker J Saggau W Sack FU Cremer J Huth C Braccio M Musumeci F Koerfer R Efficacy and safety of very low-dose self-management of oral anticoagulation in patients with mechanical heart valve replacement Ann Thorac Surg 2010 9 1487 93 10.1016/j.athoracsur.2010.06.069 20971245

[B12] Acar J Iung B Boissel JP Samama MM Michel PL Teppe JP Pony JC Breton HL Thomas D Isnard R de Gevigney G Viguier E Sfihi A Hanania G Ghannem M Mirode A Nemoz C AREVA: multicenter randomized comparison of low-dose versus standard-dose anticoagulation in patients with mechanical prosthetic heart valves Circulation 1996 9 2107 12 10.1161/01.CIR.94.9.2107 8901659

[B13] Chesebro JH Knatterud G Roberts R Borer J Cohen LS Dalen J Dodge HT Francis CK Hillis D Ludbrook P Thrombolysis in Myocardial Infarction (TIMI) Trial, phase I: a comparison between intravenous tissue plasminogen activator and intravenous streptokinase: clinical findings through hospital discharge Circulation 1987 9 142 54 10.1161/01.CIR.76.1.142 3109764

[B14] Stein PD Alpert JS Copeland J Dalen JE Goldman S Turpie AG Antithrombotic therapy in patients with mechanical and biological prosthetic heart valves Chest 1995 9 371S 379S 10.1378/chest.108.4_Supplement.371S 7555190

